# COPD in Primary Care

**Published:** 2012-03-30

**Authors:** Nina Szczygiel

**Affiliations:** GOVCOPP-DEGEI, University of Aveiro, Portugal E-mail: nina.szczygiel@ua.pt

‘COPD in Primary Care’ is a compilation of essential knowledge on chronic obstructive pulmonary disease as a health condition and its treatment within the primary care setting in the UK. COPD is a chronic condition that causes progressive obstruction of the airflows increasing continuously shortness of breath and is, by definition, irreversible. Anita Sharma, an enthusiastic, practicing general practitioner believes that primary care can have crucial impact on managing this potentially fatal condition. In a clear, concise way the author aspires to drive health care professionals into effective and quality care, according to the National Institute for Health and Clinical Excellence (NICE) guidelines and the recent General Medical Services (GMS) contract.

The book contains in total 24 chapters. The first 21 rather brief chapters give a reader a general overview of COPD as a chronic condition and the importance of the primary care setting for its structured managing. Chapter 1 defines the chronic obstructive pulmonary disease and points out differences between four major international thoracic societies in the classification of its severity. Chapter 2 portrays the prevalence, mortality and the burden of the disease on the general practice. COPD is a leading cause of mortality and morbidity in the world and, due to its character, it puts both, organisational and financial pressure to already overburdened care systems. COPD is currently part of the Quality and Outcomes Framework in the UK, which has brought some positive perspectives on impact that primary care can offer in managing this disease. Chapters 3 and 4 depict pathophysiology and risk factors. In Chapter 5 we find major developments in the national profile of COPD, which resulted in having well-defined and clear guidelines for managing this condition. Diagnosis, disease severity, spirometry, as the essential test of assessing lung function, and pulse oximetry, as a method of assessing arterial oxygen saturation, are topics of Chapters 6–9. Chapter 10 encompasses the general question on how to manage the disease, including some very interesting practical matters, such as ‘who can and how to order oxygen’. Smoking remains the principal cause of COPD and smoking cessation has been on the top of the health agenda for years. Chapter 11 points out the improvement in quality of life of patients who quitted smoking and a challenge of making that happen by presenting two models of smoking cessation used in primary care. In Chapters 11–14 we can find practical information on acute exacerbation, avoiding hospital admission and self-managing the disease. Some interesting remarks on empowering patients to take health into their own hands can be found here. The following chapter discusses the impact COPD exerts on caregivers and calls for involving them in the care plan and ongoing case management. Chapter 16 lists secondary care referral indicators. Chapter 17 specifies the concept of pulmonary rehabilitation: its availability, goals and components. The next two chapters picture issues related with palliative care in COPD and practice-based commissioning, the second one being a multidisciplinary approach providing a range of services under the umbrella of the primary care team and promising earlier discharge and reduced hospital admissions. As a result of the NHS initiative of developing General Practitioner with a Special Interest (GPwSI) clinics, over 4000 of such entities have been established up to today. As they focus on less complicated cases in primary care, more attention and time can be given to patients with serious conditions. This policy and its implications are thoroughly discussed in Chapter 20. It is followed by ideas on clinical audit (Chapter 21). Chapter 22 is a set of case studies that represent situations most commonly present to the GP surgery. Chapters 23 and 24 contain a collection of multiple choice questions allowing the reader testing the knowledge. An excellent idea! Additionally, a glossary of terms in respiratory medicine is included. 

‘COPD in Primary Care’ aims at bringing to light information relevant to primary care professionals: general practitioners and primary healthcare teams about the diagnosis and treatment of COPD in the primary care setting. I would, however, affirm that this book can be appealing not only to care environment agents, but to researchers and any regular healthcare system user as well. Health professionals can perhaps find initial chapters quite obvious. For patients at an early stage of the disease and their carers it can be first, compulsory reading in order to obtain a summary of relevant issues related with COPD.

The biggest advantage of this book is its conciseness and an indisputable clarity of the language. Terms are well explained and each chapter includes a key messages box summing up the most relevant findings. The book will surely not substitute the compendium of knowledge for medical specialists, and it does not aim to do so, but it states the problem well and opens the door for further reading. For those who intend deepening their knowledge, there are references at the end of each chapter.

The book gives a broad perspective toward several aspects of care in COPD, although it does not mention the concept of integrated care as such. The focus is given on the primary care setting as the one that can provide early and accurate diagnosis and is able to ensure an effective follow-up after an eventual specialist care intervention.

Though it is not an international position, with the UK legislation on the basis of practical information and recommendations, it gives an interesting perspective into the role of primary care in COPD, and is definitely worth reading. I would like to give it 4 stars out of five.

Dear IJIC Reader, if you have an interest in learning how primary care can contribute to providing effective, patient-centered and quality health care for patients living with COPD, this book is surely for you!

